# The chronic challenge—new vistas on long-term multisite contacts to the central nervous system

**DOI:** 10.3389/fneng.2015.00003

**Published:** 2015-03-18

**Authors:** Ulrich G. Hofmann, Jürgen Krüger

**Affiliations:** ^1^Section for Neuroelectronic Systems, Clinic for Neurosurgery, Albert-Ludwigs-University FreiburgFreiburg, Germany; ^2^Cluster of Excellence “BrainLinks-BrainTools” EXC 1086Freiburg, Germany; ^3^AG Hirnforschung, Universität FreiburgFreiburg, Germany

**Keywords:** multisite neuronal recording, gliosis, indwelling implants, compliance match hypothesis, flexible microprobes, hydrogel coating, polymeric microelectrodes

This Special Research Topic at hand is a collection of contributions from eminent research groups shedding light on several aspects of the still unresolved problem of a truly chronic cortical interface to enable long term brain-machine interfacing to human patients.

The hypothesis article of Fernandez et al. (2014) adds to the three generally agreed-on features for biocompatibility (bio-safety, bio-stability, and bio-functionality) with a fourth one that mirrors the demand for “bio-tolerability.” Sommakia et al. (2014a) study aims to reduce the almost-immediate adsorption of non-cellular tissue components upon insertion by dip-coating polyethylene glycol (PEG) as a “stealth” cover. It points toward a beneficial alteration of adsorption on the probe, but cautions PEG's immediate use for long term implants in the brain. In fact, in a second contribution based on a mixed-brain culture (Sommakia et al., 2014b), they show evidence for a complex response of glia cells on micro-wires dip-coated with PEG and/or lipopolysaccharides (LPS), but not of neurons, which is somewhat contradictory to pure *in vivo* findings.

In the same context of passive probe coatings, De Faveri et al. (2014) moved toward a more “natural” method by coating glass-insulated micro-wires with fibrin hydrogel, as a biological cushion between brain and probe. Using immunofluorescence techniques, they were able to demonstrate a beneficial effect on longer term astrocytic responses and successful encapsulation of brain cells in fibrin as in Richter (2012).

Beyond the modulatory effects of passive surface coatings, two articles review organic coatings for micro-contacts in the nervous system. The contribution by Asplund et al. (2014) concisely reviews electrodes based on conductive polymers, not only for improving site-tissue coupling, but also for electrically eluting anti-inflammatory drugs using various stimulation patterns. They go to great depth on how to apply this elution process to a living being, since the active elution technique inherently requires compliance with demanding bio-compatibility issues.

The review of Aregueta-Robles et al. (2014) addresses the topic of organic and nanoscopic coatings with a wider perspective, and thus provides an excellent overview regarding a huge variety of the various reported approaches including the “living electrode” of Ochiai et al. (1980) and their adaptation by Richter et al. (2010, 2011).

Extending the time frame of all above mentioned studies, Prasad et al. (2014) investigate whether the brain's foreign body response is the sole cause for poor electrode yield using Pt/Ir micro wire arrays. They state that leading aspects include the suboptimal construction of the micro wires, as well as the severing of the blood brain barrier upon insertion. In order to achieve a deeper insight into the suboptimal micro array construction, another study of the same group (Sankar et al., 2014) analyses long-term impedance spectra using FEM simulation, and concludes that the initial increase in electrode-tissue impedance *in vivo* can be attributed to cell attachment and gliosis on the micro wires. Furthermore they show that the long-term decrease is probably caused by de-lamination and cracks in the wire's insulation layer.

Even though the contribution of Castagnola et al. (2014) discusses the use of nano materials for improving the signal quality from brain micro-recordings as well, its main theme is the interpretation of the so called “compliance match hypothesis” (Stieglitz and Meyer, 1999) by making cortical interfaces softer reducing the permanent mismatch between rigid micro probes and the brain's softness. Obviously, comparing elastic moduli of brain and probe materials reveals a discrepancy of several orders of magnitude, and so the positive evidence of De Faveri et al. (2014) may be based on a compliance adaptation between both due to the fibrin layer. However, Castagnola et al. (2014) as well as Richter et al. (2013), Sohal et al. (2014) and Xie et al. (2014) all use substrate materials for their multisite arrays, which are still far away from brain's bulk modulus. More importantly, they are flexible (“soft” when compared to brain tissue) due to their geometrical setup. We hypothesize that this flexibility might be the reason for Krüger's 7 year recording record in the non-human primate using ultra-thin metal wires (Krüger et al., 2010). Castagnola et al. (2014) approaches a similar goal by electro-depositing microscopic spheres at the working end of very thin wires—intended for subarachnoidal recordings. Sohal et al. (2014) in contrast, intracortically implants a geometrically “wavy” structure and shows evidence for good recordings from a limited number of animals for up to 2 years. The histological findings from their and Richter's thin multisite implants depict a reduced gliosis, and thus corroborates the need for a matching compliance and intracortical softness. Richter's probes are made from polyimide (Rubehn et al., 2010), and they explain in detail how to implant them with help of a simple removable support.

This removable support in turn provides additional value, as Xie et al. (2014) state. Their use of label-free, endoscopic, *in vivo* Optical Coherence Tomography by the very same fiber supporting implantation demonstrates the first optical online monitoring of deep indwelling processes. This method may provide new vistas on the traditional problem of foreign body response, and may therefore shorten the time we have to spend in the Trough of Disillusionment.

Whatever effort is required, and whichever ideas will finally prove optimal for reaching the Plateau of Productivity for brain-machine interfaces, we are thankful that the need for bio-tolerability and reliability of brain implants over a substantial period of time, has already sunk into the community. Eleryan etal.'s (2014) single neuronal signal tracking method demonstrates this spirit of pro-active optimism by providing a highly valuable algorithm for following the progress of an individual neuron's participation in information processing.

This Special Research Topic's collection won't be the final word with respect to chronic implants, but for now it sets a roadsign toward truly stable neuro-cortical interfaces that lie on the horizon.

## Conflict of interest statement

The authors declare that the research was conducted in the absence of any commercial or financial relationships that could be construed as a potential conflict of interest.
